# From Bench to Chairside: Collagen Scaffolds in Combination with Mesenchymal Stromal Cells for Gingival Augmentation

**DOI:** 10.3390/dj13100460

**Published:** 2025-10-08

**Authors:** Polina Koteneva, Nastasia Kosheleva, Alexey Fayzullin, Yana Khristidis, Egor Bunin, Yuri Efremov, Diana Zhukova, Sergey Tkachev, Aida Kulova, Timur Rasulov, Anna Vedyaeva, Tatiana Brailovskaya, Peter Timashev

**Affiliations:** 1 Institute for Regenerative Medicine, Sechenov University, 119991 Moscow, Russia; 2 Central Research Institute of Dentistry and Maxillofacial Surgery, 119991 Moscow, Russia; 3 E.V. Borovsky Institute of Dentistry, Sechenov University, 119991 Moscow, Russia

**Keywords:** gingiva soft tissue augmentation, angiogenesis, vascularization, MSC, mesenchymal stromal cells, xenogenic materials, collagen scaffolds, Mucoderm, Fibro-Gide, FibroMATRIX, dentistry, regenerative medicine

## Abstract

**Background/Objectives:** Gingival tissue deficiencies present significant treatment challenges. We investigated three xenogeneic collagen scaffolds—Fibro-Gide, FibroMATRIX, and Mucoderm—with and without human gingival MSCs for soft tissue augmentation. **Methods:** The study assessed scaffold properties (mechanical properties and micro-CT structure), cytocompatibility, ex vivo vascular growth stimulation (CAM-test), and in vivo effects in rabbit model. **Results:** All scaffolds were cytocompatible and maintained MSC viability via extract and contact cytotoxicity tests. Fibro-Gide showed the highest porosity at 78.5%, followed by FibroMATRIX at 64.3%, while Mucoderm had the lowest porosity at 33.2%. Mucoderm exhibited the greatest stiffness due to its dense structure, contrasting with the more similar mechanical properties of Fibro-Gide and FibroMATRIX. In an ex vivo HET-Cam model of the angiogenic response, Fibro-Gide exhibited reduced blood vessel length and blood flow rate compared to FibroMATRIX and Mucoderm. In vivo, Mucoderm resorbed completely, FibroMATRIX demonstrated optimal partial degradation, and Fibro-Gide retained most of its collagen structure. **Conclusions:** The FibroMATRIX with MSCs combination showed particularly promising results for enhancing tissue thickness and vascularization, suggesting this approach could significantly improve gingival regeneration outcomes.

## 1. Introduction

Regeneration of the oral tissue, particularly the gingival mucosa, is an integral part of any dental surgeries, which is increasing due to the growing needs of medical applications. Tissues regeneration necessity is conditioned by a surgical intervention for the removal of defective tissues or the harvesting a tissue for transplantation, for example, a free gingival graft [1]. Moreover, sometimes it is necessary to grow an oral tissue due to progressive gingival recession. Thus, one of the most important issues in this field is how to make soft tissue regeneration quicker and less painful for patients. It is crucial for the improvement of patients’ well-being to decrease post-surgical complications and incrementally improve the chances of successful operations [2].

In this regard, a promising way of enhancing regeneration is the application of collagen-based materials, such as membranes or sponges. These materials have significant advantages: (1) simplicity of production and surgical application; (2) low immunogenicity; (3) in addition to becoming a scaffold for a newly formed tissue, they improve the mechanical protection of a wound [3]. Recent studies show that there is a problem in this approach: a low level of vascularization of the newly formed tissues, especially in treatment, including membrane usage [4]. The 3D structure of sponge-shaped materials could provide the required space for angiogenesis. Moreover, the wide diversity of material formation and modification techniques present materials with different structure, such as porosity and possible degradation pace [5,6]. Additionally, it is known that porosity changes could affect cell adhesion and proliferation [7,8].

Although collagen-based materials have a number of advantages, it is worth noting that their healing effect on soft tissues may not cover all the details as one might hope. By themselves, collagen materials are not able to control inflammation at the implantation site or stimulate a tissue response towards regeneration. Mesenchymal stromal cells (MSCs) and their derivatives can help eliminate this limitation more effectively. MSCs are adult stem multipotent mesenchymal stromal cells, which are involved in tissue regeneration processes [9,10]. It could be easily harvested from different tissue sources of patients or healthy donors, including gingiva, fat, bone marrow, and dental pulp [11,12,13,14,15]. It is reliably known that most of their impact on cells and tissues is achieved thanks to the paracrine effect—the ability to induce cells respond by excreting the cocktail of biologically active substances comprising cytokines, chemokines, extracellular vesicles, microRNAs, and others [16,17]. These factors are responsible for cell proliferation, angiogenesis, inflammation regulation, tissue regeneration, immunomodulation, and much more, which allows them to show astonishing results in cell-based regenerative therapies [18,19]. Nevertheless, this type of therapy is also not an ultimate solution, as MSCs are hard to attach to the wound and can be easily be re-damaged during the post-operative period.

In this work, we tried to combine collagen-based sponge-shaped materials with mesenchymal stromal cells to enhance the regenerative properties of the final product and eliminate the shortcomings of each therapy application separately. We compared the structure and mechanical properties of each material and their biocompatibility to identify how these parameters could reflect on the in vivo degradation pace and the MSCs’ effects for regeneration. However, the collagen-based materials combined with the MSCs have not yet been widely tested in preclinical and especially clinical trials, and we believe that it is a promising technique due to its potential to enhance gingival augmentation outcomes by promoting tissue regeneration, improving biocompatibility, and facilitating the healing process.

## 2. Materials and Methods

### 2.1. Fibro-Gide, FibroMATRIX, and Mucoderm Materials

Mucoderm is a volumetric, resorbable soft tissue graft made from porcine skin (Botiss, Zossen, Germany). Fibro-Gide (Geistlich, Wolhusen, Switzerland) is a porcine, porous, resorbable, and volume-stable collagen matrix. FibroMATRIX (Cardioplant, Penza, Russia) is a mechanically durable, resorbable, volumetric scaffold without shrinkage after transplantation made of animal collagen. For in vitro extract cytotoxicity experiments, we used 0.1–0.2 g of each material, and for the rest of the investigations, including in vivo experiments, we used pieces that were 0.5 cm^2^ (0.7 × 0.7 cm).

### 2.2. Micro-CT Scaffolds Preparation, Visualization, and Analysis

#### 2.2.1. Sample Preparation

Briefly, prior to micro-CT investigation, samples were placed in 3% phosphotungstic acid, dissolved in distilled water for 24 h, and kept on the rotary shaker at room temperature. After staining, samples were washed and stored in distilled water at room temperature.

#### 2.2.2. Micro-CT Imaging

A plastic tube filled with distilled water containing the contrasted sample was placed on the sample holder in a SkyScan 1276 micro-CT (Bruker, Kontich, Belgium) and scanned using the following parameters: AlCu filter, 90 kV source voltage, and 200 μA source current. Sample was scanned with the 6 μm voxel resolution. The rotation was set to 180° around the vertical axis of the sample, with two middle frames for each 0.2° angle step.

#### 2.2.3. Micro-CT Image Analysis

After scanning, the data were reconstructed using Bruker’s NRecon software (version 1.6.9). During reconstruction, the ring artifact reduction value was set to 20% and the beam hardening correction value to 30%.

Multiple methods were used to visualize the reconstructed micro-CT images. Two-dimensional visualizations were performed using the open-source software 3D Slicer (version 5.8.1). Segmentation and subsequent porosity analyses were carried out using Bruker CTAn software (version 1.17.7.2), and the resulting segmented images were visualized with Bruker CTVox (version 3.3.0). Porosity analysis was conducted using Bruker CTAn. First, a small rectangle was defined around the scaffold boundary, and this region of interest (ROI) was exported as a series of 8-bit BMP images. ROI volumes were varied in size; to enable a comparison of measurements, the volumetric data were normalized to a constant volume of 1 mm^3^ before analysis. A series of filters was then applied to the image stack in CTAn for segmentation and preprocessing (filtering, thresholding via the Riddler–Calvard algorithm, and ROI shrink-wrap). Finally, 3D analyses provided porosity information in spreadsheet format and generated a 3D model for further visualization in CTVox.

### 2.3. Microindentation

The microindentation measurements were performed using a MicroTester G2 micro-scale mechanical test system (CellScale, Waterloo, ON, Canada) in the displacement–control mode. Wet samples incubated in PBS for 2 h were placed on the hard sample stage. The used indenter probes had a stiffness of 12 N/m and a spherical tip with a radius of 500 μm. Indentations were performed up to the depth of 300–400 μm with the indentation speed of 30 μm/s, the force relaxation was recorded for 30 s before the probe retraction. At least five indentations were performed in different places over each sponge. The load–displacement curves were processed to obtain the effective Young’s (elastic) modulus and the viscoelastic parameters of the standard linear solid model (SLS) using previously developed MATLAB (9.0 R2016a, MathWorks, Natick, MA, USA) code [20]. The loading part of a curve was processed with Hertz’s model to obtain Young’s modulus:

(1) $$F\left(\delta \right)=\frac{4\sqrt{R}}{3(1-\nu^{2})}f_{BEC}\left(\delta \right)E\delta^{\frac{3}{2}};$$

where *F* is the force acting on the cantilever tip; δ is the indentation depth; ν is the Poisson’s ratio of the sample (assumed to be time-independent and equal to 0.5); *R* is the radius of the indenter; and $f_{BEC}\left(\delta \right)$ is the finite thickness (bottom-effect) correction function. The finite thickness correction was applied since the indentation depth was comparable with the sample thickness (~1 mm for Mucoderm). For the viscoelastic processing of indentation–relaxation data, we used the Lee–Radock model [21]:

(2) $$F\left(t,\;\delta (t)\right)=\frac{4\sqrt{R}}{3\left(1-\nu^{2}\right)}\int_{0}^{t}f_{BEC}\left(\delta \right)E(t-\xi )\frac{d\delta^{\frac{3}{2}}}{d\xi }d\xi ;$$

where *t* is the time initiated at the contact; ξ is the dummy time variable required for the integration; and *E*(*t*) is Young’s relaxation modulus for the selected rheology model. A standard linear solid (SLS) model was chosen as a relaxation function:

(3) $$E\left(t\right)=E_{\infty }+(E_{0}-E_{\infty })e^{-\frac{t}{\tau }}$$

where the main parameters are: instantaneous Young’s modulus $E_{0}$, long-term Young’s modulus $E_{\infty }$, and relaxation time τ.

### 2.4. Cytocompatibility

The biocompatibility of Fibro-Gide, FibroMATRIX, and Mucoderm was assessed through extract cytotoxicity tests utilizing the AlamarBlue assays. Furthermore, MSCs were cultured on these materials to analyze contact cytotoxicity using the Live/Dead assay.

For the biocompactibility assessment the primary culture of human gingival mesenchymal stromal cells was used. MSCs were obtained from the Biobank of Sechenov University (Moscow, Russia) and were cultured at 37 °C, 95% humidity, and 5% CO_2_.

Briefly, cells were usually examined using a phase-contrast microscope Axio Vert A1 (Carl Zeiss, München, Germany), with the medium replaced every 3 days.

#### 2.4.1. Extract Cytotoxicity

We performed the AlamarBlue assay to assess the biocompatibility of Fibro-Gide, FibroMATRIX, and Mucoderm. MSCs were seeded in 96-well plates (Corning, Glendale, AZ, USA) at a density of 5000 cells per well. The extracts were prepared as described elsewhere [22]. Briefly, 0.1–0.2 g of each material was incubated with the MSC growth medium at 37 °C, 95% humidity, and 5% CO_2_ for 24 h. Serial dilutions from 100% to 0.39% extract were then prepared and added to the MSCs in triplicate for each concentration point. Sodium dodecyl sulfate (SDS) dilutions served as a positive control, starting at a concentration of 1.5 mg/mL, while the MSC growth medium served as a negative control (0% extracts). The cells were incubated with the extracts, SDS, and growth medium for 24 h under standard conditions.

The extracts, SDS dilutions (positive control) and growth medium (negative control) after 24 h of incubation were replaced with AlamarBlue cell viability reagent (Invitrogen, Waltham, MA, USA) diluted 1:10 with Dulbecco’s Modified Eagle’s Medium (DMEM)/F12 (1:1, Biolot, St. Petersburg, Russia) according to the manufacturer’s protocol. The cells were incubated for 2 h, then the plates were read on a Victor Nivo spectrofluorometer (PerkinElmer, Waltham, MA, USA) at an excitation wavelength of 580/20 nm and an emission wavelength of 625/30 nm.

#### 2.4.2. Viability (Live/Dead) Analysis

The Fibro-Gide, FibroMATRIX, and Mucoderm scaffolds were seeded with MSCs and cultured under standard conditions (37 °C, 95% humidity, 5% CO_2_) for three days. A live/dead assay (calcein-AM, Sigma-Aldrich, Burlington, MA, USA; propidium iodide, Thermo Fisher Scientific, Waltham, MA, USA; Hoechst 33285, Thermo Fisher Scientific, Waltham, MA, USA) was performed on the third day using a concentration of the reagents according to the manufacturer’s protocol; live cells were stained green (calcein-AM), dead cells were stained red (propidium iodide), and nuclei were additionally stained blue (Hoechst 33285). Visualization was performed using an EVOS M5000 Imaging System (Thermo Fisher Scientific, Waltham, MA, USA).

### 2.5. Scanning Electron Microscopy

The Fibro-Gide, FibroMATRIX, and Mucoderm scaffolds were seeded with MSCs and cultured under standard conditions (37 °C, 95% humidity, 5% CO_2_) for three days; both with and without cells, scaffolds were prepared for scanning electron microscopy (SEM) analysis. Initially, the samples were fixed in a 3% glutaraldehyde solution in phosphate-buffered saline (PBS) overnight at +4 °C. Following fixation, the samples were washed three times with PBS, each wash lasting 5 min. The matrices were then subjected to a secondary fixation in a 1% osmium tetroxide (OsO_4_) solution in PBS for 40 min at room temperature. Afterward, both cell-seeded and cell-free samples underwent three additional washes with PBS. Dehydration was achieved through a graded series of ethanol washes: twice with 50% ethanol for 5 min each, twice with 70% ethanol for 10 min each, once with 80% ethanol for 5 min, and twice with 95% ethanol for 5 min each, followed by two washes with acetone for 5 min each. The matrices were dried using critical point drying, coated with gold under vacuum conditions, and examined with a CamScan-S2 scanning electron microscope (Cambridge Instruments, Cambridge, UK) to analyze the replicas.

### 2.6. Ex Vivo HET-CAM—Hen’s Egg Test on Chorioallantoic Membrane

Fertilized chicken eggs (*Gallus gallus*) were obtained from a poultry farm and transported under controlled temperature conditions to ensure viability and quality. Before the experiment, the eggs were stored in a thermostat at 10 °C. Incubation was carried out at 37 ± 0.5 °C with a relative humidity of 65 ± 5% in a horizontal position using an automatic incubator.

On the third day of embryonic development, the eggshell was disinfected, and a window was created at the blunt end of the egg. The membranes were carefully removed using forceps, and 3 mL of albumen was extracted with a syringe. The embryos were then incubated in a stationary vertical position until day 7.

On the seventh day, sterilized matrices were placed onto the chorioallantoic membrane (CAM). The implantation site was selected according to the key criteria of CAM analysis—midway between the embryo and the outer boundary, positioned between two major blood vessels. The number of analyzed samples was as follows for different groups: Control: *n* = 9, Fibro-Gide: *n* = 7, Mucoderm: *n* = 6, Fibro-Matrix: *n* = 6.

On the tenth day of embryonic development, results were recorded using a stereomicroscope SMZ800N (Nikon, Tokyo, Japan). The quantification was performed using ImageJ software (version 1.53e) with the Cell Counter Plugin (National Institutes of Health, Bethesda, MD, USA). The macroscopic assessment of the angiogenic response was conducted based on vessel length, vascular network area, and vascular index. The vascular index was measured as the fold difference between the number of vessels before implantation and the number of vessels 72 h after implantation in the area surrounding the matrix. Vessel length and area were expressed as the ratio of their measurements at 72 h post-implantation to those at 0 h. Pre-implantation values were set at 100%, and all data were converted into fold changes relative to this baseline.

A laser speckle imaging system (RWD Life Science, Shenzhen, China) was used to assess blood flow velocity in CAM. This method enabled real-time, non-invasive visualization of perfusion. Regions of interest (ROIs) were defined both prior to matrices implantation and 72 h post-implantation, encompassing the implantation site and an adjacent area of comparable dimensions. The acquired images were processed using dedicated software to generate perfusion curves for each region. From these data, the ROI-specific blood flow velocity values were extracted and subsequently compared between baseline (0 h) and 72 h post-implantation.

Statistical comparisons were performed using the Kruskal–Wallis test with the post hoc Dunn’s multiple comparisons, and results of *p* ≤ 0.05 (*) were considered significant with data given as median ± 5–95 percentile.

### 2.7. Animal Experiments

In this experimental study, female rabbits of the “Gray Giant” breed weighing 3000–3200 g were used, with individuals divided into six groups of individuals each. The animals were categorized into the following groups: Group 1 (Fibro-Gide), Group 2 (Fibro-Gide + MSC), Group 3 (FibroMATRIX), Group 4 (FibroMATRIX + MSC), Group 5 (Mucoderm), and Group 6 (Mucoderm + MSC). The effectiveness of gingival soft tissue restoration was compared after closed gingival mucosa augmentation with implantation of the materials and matrices with MSCs cultured for 3 days under the flap in rabbits of the breed. Surgical intervention was carried out targeting the upper jaw on both sides of the vestibular surface of the gingival mucosa. A linear incision was made, followed by apical movement and fixation of the superficial flap with sutures. The examined material was placed on the periosteum and secured with double nodular sutures, depending on the group assignment. All manipulations were performed under conditions of medication analgesia using a solution of tiletamine and zolazepam (ZOLETIL 100) and a solution of medetomidine (Meditin). Before surgery, animals were anesthetized intramuscularly with combined anesthesia ZOLETIL 100 (VIRBAC, Carros, France) at a dosage of 15 mg/kg and Meditin (Apicenna, Moscow, Russia) at a dosage of 0.5 mg/kg. After the 14th day, the rabbits were euthanized by an injection of a solution of ZOLETIL 100 at a dosage of 60 mg/kg. The sites of implantation were dissected together with 2–3 mm of surrounding tissues. In order to investigate the systemic effects of the implants, a kidney, a liver, and a complex of heart and lungs were collected from all animals. The tissues and organs were fixed in neutral buffered formalin for 24–48 h.

### 2.8. Histological and Morphometric Analysis

Four-μm-thick sections of the formalin-fixed-paraffin-embedded tissue samples were stained with hematoxylin and eosin (H&E); and with Picrosirius red for the detection of collagen fibers. A LEICA DM4000 B LED microscope, equipped with a LEICA DFC7000 T digital camera running under the LAS V4.8 software (Leica Microsystems, Wetzlar, Ger-many) was used for the examination and imaging of the samples.

For immunohistochemical analysis, four-μm-thick sections of the formalin-fixed-paraffin-embedded tissue samples were deparaffinized, incubated with 3% hydrogen peroxide (H_2_O_2_) for 10 min, underwent heat-induced epitope retrieval (pH 6.0 sodium citrate buffer, 30 min in 80 °C water bath), additionally blocked with background block (Cell Marque, Rocklin, CA, USA), incubated with mouse monoclonal primary antibodies against α-smooth muscle actin (α-SMA) (A2547, Merck Millipore, Burlington, MA, USA, diluted 1:400), and detected by HRP-conjugated secondary goat antibodies (G-21040, Invitrogen, Carlsbad, CA, USA, diluted 1:1000) and diaminobenzidine (DAB) with hematoxylin counterstaining.

Blood vessel density was evaluated at ×200 magnification in 10 representative fields of view. The results of the blood vessel density analysis were counted as an average number per 1 mm^2^ for each sample. The thickness of the gingival submucosa was evaluated as an average of five measurements at a distance of 400 μm from each other. Cell counting and implant area measurement were performed on histological slides stained with Picrosirius red, which were digitized on a Hamamatsu Nanozoomer S20 histological scanner (Hamamatsu Photonics, Hamamatsu, Japan) at ×40 magnification. Scanned images were analyzed in QuPath version 0.4.3. Square regions in the centers of implantation sites with an area of 1 mm^2^ were selected, in which cell detection was performed using the StarDist model version 0.7.3. The StarDist operating parameters were threshold = 0.5, cellExpansion = 5.0, cellConstrainScale = 1.5. The relative area of membrane material was measured in the same regions by isolating and segmenting the red pixels (indicating collagen stained with Picrosirius red) using ImageJ software (version 1.54f), dividing the obtained area by the area of the field of view and multiplying by 100%.

The statistical analysis of the experimental data was performed with a standard program package, GraphPad Prism version 8.00 for Windows (GraphPad Software, Inc., San Diego, CA, USA). The intergroup differences were analyzed by the one-way ANOVA followed by Tukey’s multiple comparison test. The statistical analysis results were presented as column graphs of the mean values and standard deviations (SD). *p*-values equal or less than 0.05 were considered statistically significant.

## 3. Results

### 3.1. Micro-CT Visualization and Porosity Analysis of Collagen Scaffolds

**Figure 1:**
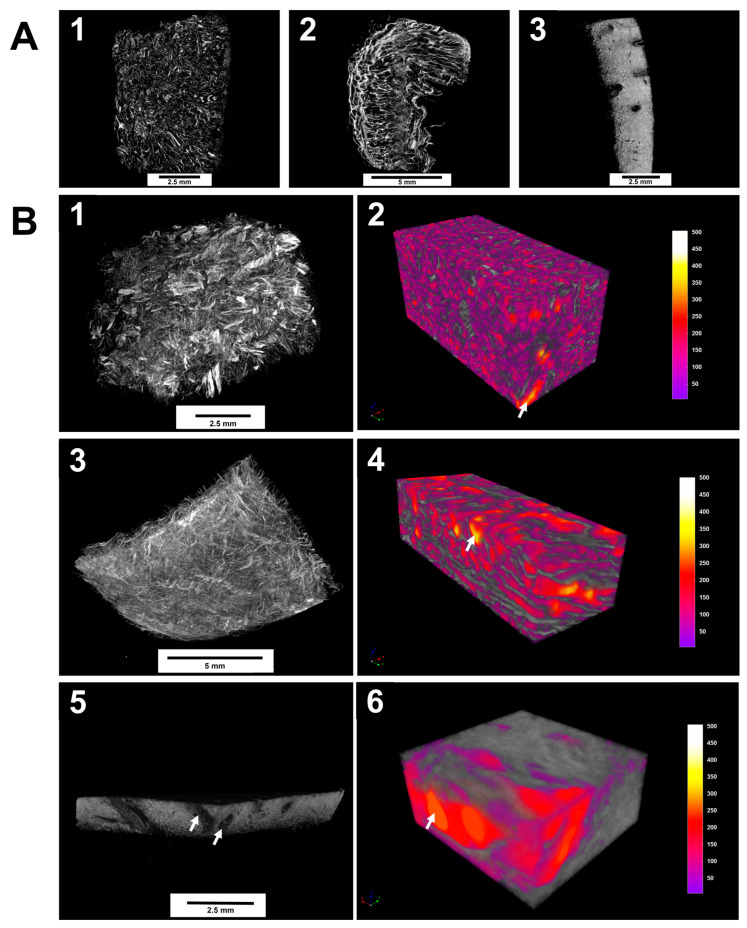
Micro-CT visualization of the Fibro-Gide, FibroMATRIX and Mucoderm. (**A**) Two-dimensional visualization of the imaged samples in grayscale obtained with the ROI (region of interest) tool; (**1**–**3**) Longitudinal section of the Fibro-Gide, FibroMATRIX, and Mucoderm matrices, respectively; (**B**) Three-dimensional visualization of the imaged samples in grayscale and different approaches for the image analysis. (**1**,**3**,**5**) Volume-rendering model of the Fibro-Gide, FibroMATRIX and Mucoderm, respectively. (**2**,**4**,**6**) Porosity analysis of the Fibro-Gide, FibroMATRIX, and Mucoderm with the segmentation of the pores (color-coded depending on pore volume) and fibers (gray). White arrows indicate pores in the materials.

All matrices exhibited a porous structure, which can potentially enable the ingrowth of cells after grafting. We found significant differences in the pore size and surface morphology of the porous scaffolds. Obtained 2D-images of the internal structure indicate that Fibro-Gide has been the matrix in which the pore morphology was presented better than other samples (Figure 1A). The region of interest is the structure-forming part of the scaffold, and the pore lumen is taken as the image background.

Micro-CT visualization results revealed the pore structure of the three matrices studied with pore sizes ranging from 50 to 500 μM (Figure 1B). The samples’ total volume and pore space volume were measured to calculate the porosities. During the analysis of the samples, it was found that they differed in their porosity. For Fibro-Gide, the porosity was 78.5% with a pore volume of 68 mm^3^. In the FibroMATRIX sample, the porosity was 64.3% with a pore volume of 10.7 mm^3^. In the Mucoderm sample, the porosity amounted to 33.2% with a pore volume of 0.41 mm^3^. Thus, Fibro-Gide achieved the highest volume porosity of the tested materials.

In Fibro-Gide and FibroMATRIX samples showed higher tortuosity of pore channels compared to the Mucoderm sample. The measured matrix morphology data are summarized in Table S1.

### 3.2. Mechanical Properties of the Fibro-Gide, FibroMATRIX and Mucoderm

**Figure 2:**
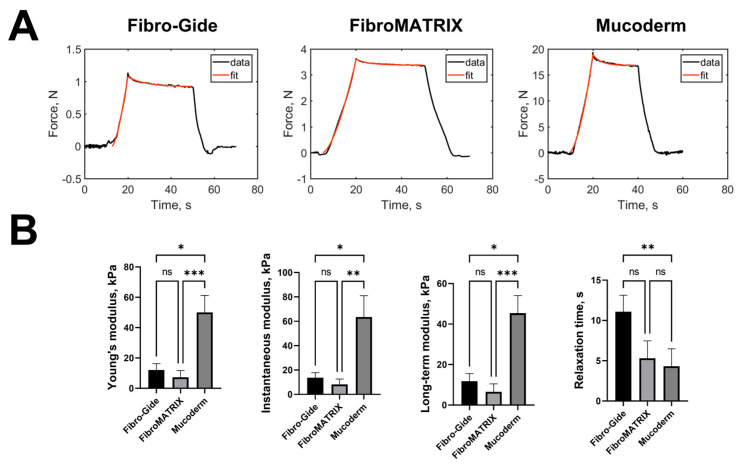
Mechanical properties of the Fibro-Gide, FibroMATRIX, and Mucoderm. (**A**) Typical indentation curves (force vs. time) for the analyzed collagen sponges together with the standard linear solid model fit (red curves); (**B**) Mechanical parameters of the collagen sponges estimated from microindentation experiments. Asterisks indicate significance in analysis for the Kruskal–Wallis test with the post hoc Dunn’s multiple comparisons test: * *p* ≤ 0.05, ** *p* ≤ 0.01, *** *p* ≤ 0.001.

The mechanical properties of collagen sponges were analyzed by microindentation experiments. The typical force curves with the viscoelastic model fits are presented in (Figure 2A). Selected viscoelastic model (SLS) described the relaxation process quite well, with a coefficient of determination R^2^ > 0.97. The measured mechanical parameters are presented in Figure 2B and Table S2.

Mucoderm sponges had the highest stiffness, all modulus values were ~four times higher than those for other sponges. This is due to the presence of a dense layer of collagen above the porous layer on both sides of the sponge and overall higher material density. Fibro-Gide sponges had slightly higher values of Young’s modulus, instantaneous and long-term moduli, and relaxation time, compared to FibroMATRIX sponges (not significantly). But the relaxation time of Fibro-Gide sponges was significantly higher than such of Mucoderm sponges, which can also be due to the dense collagen layer in the latter.

### 3.3. Biocompatibility of the Fibro-Gide, FibroMATRIX and Mucoderm

**Figure 3:**
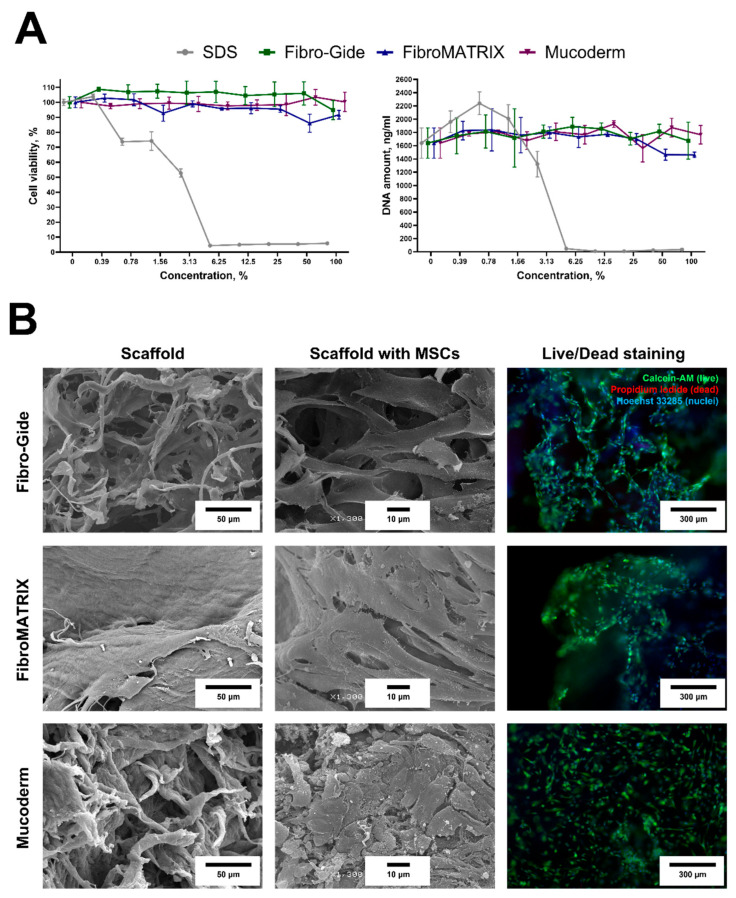
The cytocompatibility of the Fibro-Gide, FibroMATRIX, and Mucoderm materials and material surface visualization. (**A**) MSC metabolic activity (AlamarBlue assay) and DNA concentration (PicoGreen assay); sodium dodecyl sulfate (SDS) was used as a positive control; mean values ± SD. (**B**) Left column: Cell-free matrices’ surface (SEM). Middle column: MSCs cultured on the surface (3 days; SEM). Right column: Live/dead staining of MSCs cultured on the surface (3 days; calcein-AM (green, live cells), propidium iodide (red, dead cells), Hoechst 33285 (blue, nuclei).

Both Fibro-Gide and Mucoderm exhibited a fibrous structure on their surface, while FibroMATRIX had a predominantly smooth surface in the SEM images (Figure 3B). The contact cytotoxicity test did not reveal any negative effects on MSC viability and morphology. MSCs on the material surface remained viable, had spindle-shaped morphology, and covered almost the entire surface of the biomaterials. Notably, the cells did not form a dense monolayer on the Mucoderm surface, unlike the surfaces of Fibro-Gide and FibroMATRIX sponges. According to the extract test, all sponge extracts did not affect MSC viability (Figure 3A). Cell viability was more than 70% at all extract concentrations, and the DNA concentration was, on average, 1742.8 ± 155.5 ng/mL, indicating that the sponges were completely cytocompatible.

### 3.4. Vascular Response to Fibro-Gide, FibroMATRIX, and Mucoderm in the HET-CAM Model

**Figure 4:**
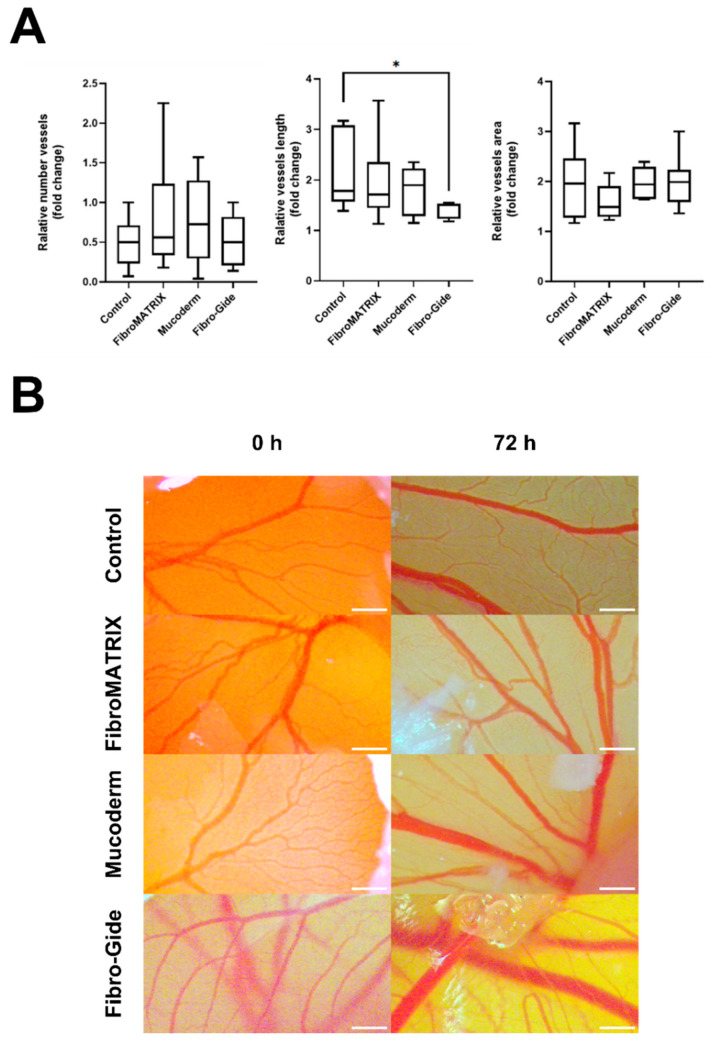
The blood vessels evaluation in ex vivo HET-CAM test for the collagen matrices. (**A**) Macroscopic evaluation of collagen matrices based on the vascular index (number vessels), vessel length, and vascular area for the control group, Fibro-Gide, FibroMATRIX, and Mucoderm. Asterisks indicate significance in analysis for the Kruskal–Wallis test with the post hoc Dunn’s multiple comparisons test: * *p* ≤ 0.05; (**B**) The blood vessels of the chorioallantoic membrane (CAM) at the implantation site at 0 h and 72 h after implantation for the control group, Fibro-Gide, FibroMATRIX, and Mucoderm; scale bar: 1 mm.

Ex vivo evaluation of the angiogenic response, based on the vascular index, vessel length, and vascular network area, demonstrated high angiogenic activity of the FibroMATRIX and Mucoderm, comparable to that of the control (Figure 4A). The macroscopic assessment confirmed the strong attachment of collagen matrices to the CAM. All collagen matrices do not have a significant impact on the development of *Gallus gallus* embryos. Seventy-two hours after implantation, no adverse effects of the FibroMATRIX and Mucoderm were observed. In embryos with Fibro-Gide, the overall vascular network length decreased 72 h after implantation compared to the control sample (Figure 4B), which may indicate a reduction in angiogenic activity in the presence of Fibro-Gide.

**Figure 5:**
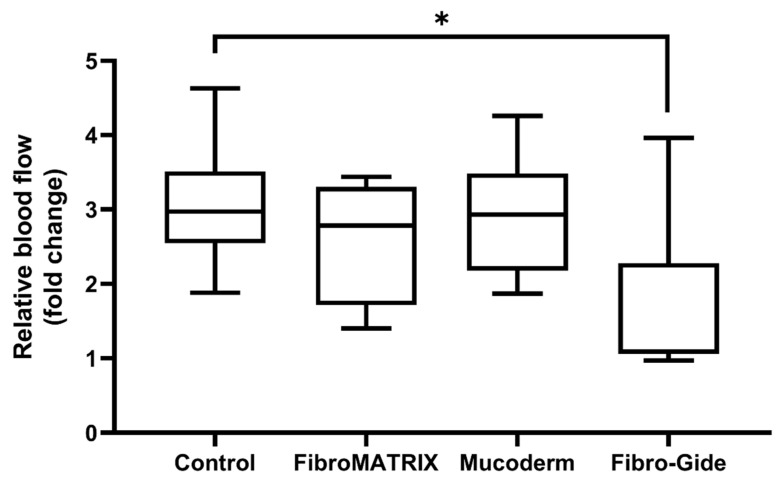
Assessment of blood flow in ex vivo HET-CAM test using the laser speckle imaging system for the collagen materials. Asterisks indicate significance in analysis for the Kruskal–Wallis test with the post hoc Dunn’s multiple comparisons test: * *p* ≤ 0.05.

The evaluation of microcirculation in the chorioallantoic membrane demonstrated good blood flow in the FibroMATRIX and Mucoderm groups compared to the control (Figure 5). At the same time, the Fibro-Gide group exhibited reduced perfusion, indicating a potential negative impact of this collagen matrix on tissue vascularization. The perfusion rate in Fibro-Gide samples increased by 3.21% at 72 h post-implantation compared to the implantation day, which is 2.96 times lower than in the control group.

The reduction in blood flow intensity in tissues in contact with Fibro-Gide may indicate an impairment of normal circulation and vascular response in this region.

### 3.5. In Vivo Studies of Fibro-Gide, FibroMATRIX, and Mucoderm Properties

**Figure 6:**
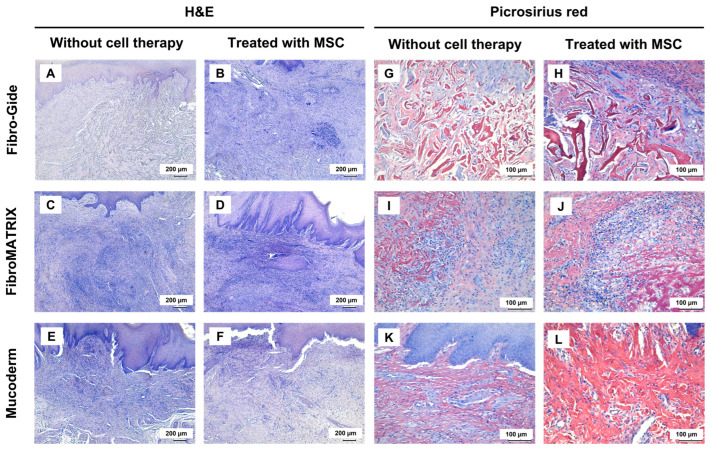
Morphological analysis of rabbit gingival tissues treated with collagen sponges with and without MSC. (**A**,**B**,**G**,**H**) morphological analysis of Fibro-Gide group; (**C**,**D**,**I**,**J**) morphological analysis of FibroMATRIX group; (**E**,**F**,**K**,**L**) morphological analysis of Mucoderm group. Hematoxylin&eosin and Picrosirius red staining; for H&E magnification ×50, scale bar 200 µm, for Picrosirius red magnification ×200, scale bar 100 µm.

**Figure 7:**
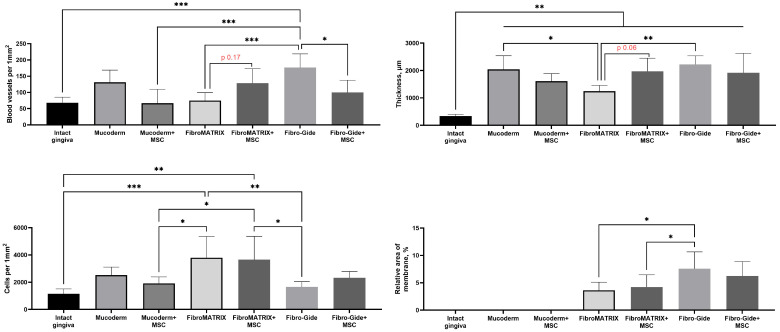
Statistical analysis of blood vessel and cell density, thickness of lamina propria and relative area of the membranes in rabbit gingival tissue. One-way ANOVA, mean values ± SD. * *p* ≤ 0.05, ** *p* ≤ 0.01, *** *p* ≤ 0.001; trend to increase blood vessels amount and tissue thickness towards FibroMATRIX and FibroMATRIX + MSC groups showed as a *p*-value with red color.

In the Fibro-Gide group, weakly resorbed spongy construct consisting of thick and dense collagen fibers was observed at the implantation site (stained red with Picrosirius red). Within this material, with a high density of thin-walled blood vessels was noted (Figure S2). Dense connective tissue formed around the implant material. The total thickness of the lamina propria in the implantation site was the highest in the experiment, largely due to the volume occupied by the membrane (Figure 6A,G and Figure S1). When using a combination of Fibro-Gide and MSCs, the tissue at the implantation site was slightly looser and there was a decrease in the density of blood vessels within the implant. The thickness of the lamina propria did not differ from that when using a membrane without MSCs (Figure 6B,H, Figure 7 and Figure S1). Immune cell infiltration in both groups with Fibro-Gide implantation was characterized predominantly by moderately dispersed macrophages accompanied by singular foreign body giant cells. Only minor foci of neutrophils were observed under the epithelium.

Residual fibers of FibroMATRIX membranes were determined at the implantation site. Their collagen nature was confirmed by the Picrosirius red staining. Inflammatory infiltration of neutrophils was observed around this material, and the area of peri-implant tissues was smaller than when using other membranes, which may indicate that inflammation prevented the development of a proliferative reaction (Figure 6C,I and Figure S1). Notably, FibroMATRIX combined with MSCs showed a tendency toward reduced inflammatory response and modest increases in both vascular density and gingival lamina propria thickness compared to scaffold-only implantation, though these differences did not reach statistical significance (Figure 6D,J, Figure S1 and Figure S2). A distinctive feature observed in both FibroMATRIX groups was a high-density immune cell infiltration, predominantly composed of neutrophils diffusely distributed throughout the entire implantation area.

A pronounced proliferative reaction was determined at the sites of implantation of Mucoderm membranes. The implant material itself was not determined and, apparently, became a provisional matrix for the fibrotic, newly formed granulation tissue. This tissue had a high vascular density and consisted of thick collagen bundles (Figure 6E,K). When using Mucoderm in combination with MSCs, the histology did not change significantly, although there was a trend towards a decrease in blood vessel density (Figure 6F,L and Figure S2). Immune cells did not form pronounced foci of infiltration; instead, individual macrophages and neutrophils were scattered between the collagen fiber bundles of the maturing granulation tissue.

Morphometric analysis of blood vessel density revealed a trend towards their increase compared to the group of the same material only for FibroMATRIX (*p* = 0.178) (Figure 7). The highest blood vessel density was observed in the Fibro-Gide group, this was the only group in which the vessel density was significantly higher than in the intact gingiva (*p* = 0.0002). However, at the same time, a significant decrease in blood vessel density was determined when combining this material with MSCs (*p* = 0.014). Morphometric analysis of the thickness of the lamina propria showed that when using all materials in combination with MSCs and without it, a significant increase in the thickness of the lamina propria was determined compared to the intact gingiva. Moreover, the increase in thickness when using Mucoderm and Fibro-Gide materials was significantly higher than when using FibroMATRIX (*p* = 0.032 and *p* = 0.004, respectively). No significant differences were found between the groups with and without MSCs for Mucoderm and Fibro-Gide, but a trend towards increased thickness was noted for the FibroMATRIX group with MSCs (*p* = 0.064).

Morphometric analysis of cell density in the implantation sites showed that the highest number of cells was observed when using the FibroMATRIX material with and without MSCs. These groups significantly differed in cell density from the intact gingiva, the Mucoderm + MSCs group and the Fibro-Gide group. These differences collaborate the observation of high density of immune cells (primarily, neutrophils) in the sites of FibroMATRIX implantation. No significant differences were found for the materials between the groups with and without MSCs.

Morphometric analysis of the relative density of the membrane material residues did not show the effect of the MSCs addition factor on the resorption rate, but it was shown that the Fibro-Gide materials occupied a larger relative area than the FibroMATRIX materials, while it was impossible to determine the presence of the Mucoderm material, since the tinctorial properties and organization of the membrane fibers did not differ from the fibers of the connective tissue formed de novo in the implantation site.

Histological evaluation of the kidney, liver, heart, and lungs of all studied animals revealed preserved architecture without pathological changes. Renal tissue showed intact glomeruli and tubules, and hepatic lobules displayed orderly hepatocyte cords without steatosis or fibrosis. Myocardial fibers and pulmonary alveoli were structurally normal, without necrosis, inflammation or edema, indicating no adverse effects from membranes or MSCs. No signs of infection were observed in any laboratory animals, including the presence of bacteria in dentinal tubules that would be indicative of periodontal disease.

## 4. Discussion

The degradation rate of collagen scaffolds significantly influences clinical outcomes in gingival augmentation procedures. Slowly resorbing matrices persist longer in tissues, impeding vascular invasion and prolonging localized inflammation, which collectively suppresses angiogenesis [23]. Conversely, rapidly degrading scaffolds may promote robust vascularization, but often at the expense of mechanical stability and volume maintenance in newly formed tissue. An optimal solution appears to be scaffolds with moderate degradation rates that align with both tissue growth and vascularization kinetics [24].

All tested scaffolds demonstrated excellent cytocompatibility, supporting MSC adhesion, spindle-shaped morphology, and viability. Among them, Mucoderm exhibited notably rapid in vivo degradation. Despite having the highest material density, stiffness, and lowest porosity (33.2%, pore area 0.41 mm^3^), it was fully resorbed. The resulting granulation tissue was rich in organized collagen bundles and blood vessels, suggesting efficient remodeling. This process could be further enhanced by MSC incorporation, as they actively promote extracellular matrix reorganization during regeneration. Furthermore, in vitro cells cultivation term also could be important to final implantation outcomes. While MSCs are actively secret enzymes (e.g., matrix metalloproteinases) that reorganize the extracellular matrix, the mechanical properties and the structure of collagen scaffolds could change during long-term cultivation [25]. The proliferation of cells also should be carefully evaluated since the cells number would also change during cultivation period.

In contrast, Fibro-Gide degraded more slowly in vivo, potentially hindering vascularization in the scaffold core and subsequent tissue formation. Both Fibro-Gide and FibroMATRIX exhibited higher porosity and greater structural tortuosity (78.5% and 64.3%, respectively) compared to Mucoderm, though Fibro-Gide was slightly stiffer. The significantly longer relaxation time of Fibro-Gide sponges—attributable to its higher tortuosity—suggests reduced diffusion efficiency, as water molecules navigate more convoluted paths. Notably, the inverse relationship between porosity and stiffness—where Mucoderm low porosity correlated with high stiffness, and Fibro-Gide high porosity correlated with lower stiffness—indicates that the mechanical properties in this system are predominantly dictated by architectural parameters rather than the base collagen material in agreement with previous study [26]. To sum up, differences in the mechanical properties likely arise from pore architecture, variations in collagen type and source and sponge formation methods.

Critically, scaffold-driven vascularization appears to depend more on initial structural properties than on granulation tissue growth rates. While studies confirm that pore size, composition, and degradation kinetics profoundly affect angiogenic potential [23,27,28], excessive porosity can compromise mechanical integrity, ultimately reducing neovascular density [29]. This was corroborated in ex vivo chorionallantoic membrane assays, where Fibro-Gide high porosity impaired angiogenic responses 72 h post-implantation. Notably, cell-seeded Fibro-Gide scaffolds showed reduced vascularization and tissue volume compared to acellular controls, possibly due to pore tortuosity limiting MSC paracrine signaling.

FibroMATRIX, however, displayed intermediate degradation, partially resorbing without excessive volume retention. In CAM assays, it maintained normal blood flow levels. Consistent with previous in vitro and ex vivo studies, MSCs promote a stronger vascular response in both CAM and Matrigel assays [30,31]. FibroMATRIX MSCs’ supplementation trended toward increased vessel density and tissue thickness at implantation sites—likely due to MSCs’ anti-inflammatory and pro-angiogenic effects [32]. Inflammation can inhibit proliferative responses near implanted materials, but MSCs may counteract this by modulating local immune balance [33].

To maximize proliferative effects, MSCs require a scaffold that provides a template for new matrix deposition. Scaffolds with moderate degradation rates optimize this balance, fostering tissue remodeling, and angiogenesis [24]. Initial scaffold thickness and volume must avoid impeding core nutrition or spatially constraining MSC effects. Central MSC localization in bulky scaffolds may attenuate paracrine signaling, diminishing therapeutic outcomes. For example, prior studies report significant angiogenic stimulation when MSCs are delivered via injection over short-term periods [33].

Our findings highlight the potential of collagen-MSC combinations for gingival augmentation, particularly in enhancing vascularization and mitigating inflammation. Despite MSCs proregenerative potential, capacity to enhance vascularization, and promote soft tissue thicknessing, the clinical translation of MSCs faces some challenges. The primary ones lie in a complex of quite lengthy and costly process of cells isolation, expansion, and characterization. In contrast, the usage of growths factors offers simpler approach, which already entered the clinical practice. However, it cannot fully replace connective tissue grafts [34] and currently only serves as a complement [35,36]. The further development of this direction, including the restoration of the periodontal tissue complex and addressing bone mass loss, may be linked to simplifying the technology and utilizing not the MSCs themselves but rather products of their secretion that possess the necessary regulatory activity [37,38,39].

However, while this study lays foundational insights, large-animal models are needed to evaluate translational feasibility for human trials. Future work should also explore patient-specific scaffolds tailored to defect size, gingival biotype, and degradation–angiogenesis coupling. Another limitation is that our study does not address periodontal disease as an infectious driver of gingival recession, where bacterial biofilms and their products can directly accelerate soft tissue and bone loss. Future models incorporating infectious etiologies will be essential to fully capture the clinical spectrum of gingival recession.

## 5. Conclusions

In this study, we systematically evaluated how scaffold degradation kinetics and volumetric properties influence new tissue formation patterns during gingival augmentation. We achieved significant soft tissue thickening through the implantation of three xenogenic collagen scaffolds, both individually and in conjunction with mesenchymal stromal cells. In vitro analyses indicated that none of the collagen sponges exhibited cytotoxic effects on gingival MSCs, demonstrating complete biocompatibility as evidenced by viability and metabolic activity assessments. In vivo observations in a rabbit model revealed distinct resorption profiles: Mucoderm underwent complete degradation, FibroMATRIX showed partial resorption, while Fibro-Gide remained largely intact. With its controlled degradation pace and favorable structural–mechanical properties, FibroMATRIX provided a supportive framework for MSCs to establish a regenerative niche, resulting in measurable improvements in both tissue volume restoration and neovascularization. MSCs enhance tissue regeneration through matrix reorganization and vascular stimulation, but their therapeutic efficacy depends on optimal spatial positioning within scaffolds to ensure effective paracrine signaling and cell–tissue interactions. These findings highlight the translational potential of combining collagen scaffolds with MSCs for clinical periodontal applications.

## Data Availability

The original contributions presented in this study are included in the article and Supplementary Materials. Further inquiries can be directed to the corresponding author.

## Supplementary Materials

The following supporting information can be downloaded at: https://www.mdpi.com/article/10.3390/dj13100460/s1, Figure S1: Morphological analysis of rabbit gingival tissues at sites of implantation of Fibro-Gide, FibroMatrix, and Mucoderm, with and without cell therapy, hematoxylin, and eosin; magnification ×200; Figure S2: Morphological analysis of rabbit gingival tissues at sites of implantation of Fibro-Gide, FibroMatrix, and Mucoderm with and without cell therapy, immunohistochemical reaction with antibodies against α-smooth muscle actin; magnification ×100; Table S1: Porosity analysis; Table S2: Mechanical parameters of the collagen sponges estimated from microindentation experiments (mean ± SD).

## References

[1] Preidl R.H.M., Reichert S., Coronel T.V., Kesting M., Wehrhan F., Schmitt C.M. (2021). Free Gingival Graft and Collagen Matrix Revascularization in an Enoral Open Wound Situation. J. Oral Maxillofac. Surg..

[2] Schmitt C.M., Moest T., Lutz R., Wehrhan F., Neukam F.W., Schlegel K.A. (2016). Long-Term Outcomes after Vestibuloplasty with a Porcine Collagen Matrix (Mucograft^®^) versus the Free Gingival Graft: A Comparative Prospective Clinical Trial. Clin. Oral Implant. Res.

[3] Shi X., Li X., Tian Y., Qu X., Zhai S., Liu Y., Jia W., Cui Y., Chu S. (2023). Physical, Mechanical, and Biological Properties of Collagen Membranes for Guided Bone Regeneration: A Comparative in Vitro Study. BMC Oral Health.

[4] Hosty L., Heatherington T., Quondamatteo F., Browne S. (2024). Extracellular Matrix-Inspired Biomaterials for Wound Healing. Mol. Biol. Rep..

[5] Rock C.A., Keeney S., Zakharchenko A., Takano H., Spiegel D.A., Krieger A.M., Ferrari G., Levy R.J. (2021). Model Studies of Advanced Glycation End Product Modification of Heterograft Biomaterials: The Effects of in Vitro Glucose, Glyoxal, and Serum Albumin on Collagen Structure and Mechanical Properties. Acta Biomater..

[6] Zhao X., Li X., Xie X., Lei J., Ge L., Yuan L., Li D., Mu C. (2020). Controlling the Pore Structure of Collagen Sponge by Adjusting the Cross-Linking Degree for Construction of Heterogeneous Double-Layer Bone Barrier Membranes. ACS Appl. Bio Mater..

[7] D’Amico E., Pierfelice T.V., Lepore S., Iezzi G., D’Arcangelo C., Piattelli A., Covani U., Petrini M. (2023). Hemostatic Collagen Sponge with High Porosity Promotes the Proliferation and Adhesion of Fibroblasts and Osteoblasts. Int. J. Mol. Sci..

[8] Kaya M.G.A., Simonca A.G., Rau I., Coman A.E., Marin M.M., Popa L., Trusca R., Dinu-Pirvu C.E., Ghica M.V. (2024). Topical Biocomposites Based on Collagen, Hyaluronic Acid and Metronidazole as Periodontitis Treatment. Pharmaceuticals.

[9] Chen M., Przyborowski M., Berthiaume F. (2009). Stem Cells for Skin Tissue Engineering and Wound Healing. Crit. Rev. Biomed. Eng..

[10] Gopalarethinam J., Nair A.P., Iyer M., Vellingiri B., Subramaniam M.D. (2023). Advantages of Mesenchymal Stem Cell over the Other Stem Cells. Acta Histochem..

[11] Musiał-Wysocka A., Kot M., Majka M. (2019). The Pros and Cons of Mesenchymal Stem Cell-Based Therapies. Cell Transpl..

[12] Tsuji W., Rubin J.P., Marra K.G. (2014). Adipose-Derived Stem Cells: Implications in Tissue Regeneration. World J. Stem Cells.

[13] Lan X., Sun Z., Chu C., Boltze J., Li S. (2019). Dental Pulp Stem Cells: An Attractive Alternative for Cell Therapy in Ischemic Stroke. Front. Neurol..

[14] Kim D., Lee A.E., Xu Q., Zhang Q., Le A.D. (2021). Gingiva-Derived Mesenchymal Stem Cells: Potential Application in Tissue Engineering and Regenerative Medicine—A Comprehensive Review. Front. Immunol..

[15] Hass R., Kasper C., Böhm S., Jacobs R. (2011). Different Populations and Sources of Human Mesenchymal Stem Cells (MSC): A Comparison of Adult and Neonatal Tissue-Derived MSC. Cell Commun. Signal..

[16] Aitzetmüller M.M., Brett E.A., Sauter M., Duscher D. (2019). Basic Principles and Current Approach for Soft Tissue Regeneration. Regen. Med. Plast. Surg..

[17] Linero I., Chaparro O. (2014). Paracrine Effect of Mesenchymal Stem Cells Derived from Human Adipose Tissue in Bone Regeneration. PLoS ONE.

[18] Peshkova M., Korneev A., Suleimanov S., Vlasova I.I., Svistunov A., Kosheleva N., Timashev P. (2023). MSCs’ Conditioned Media Cytokine and Growth Factor Profiles and Their Impact on Macrophage Polarization. Stem Cell Res. Ther..

[19] Gnecchi M., Danieli P., Malpasso G., Ciuffreda M.C. (2016). Paracrine Mechanisms of Mesenchymal Stem Cells in Tissue Repair. Methods Mol. Biol..

[20] Efremov Y.M., Kotova S.L., Timashev P.S. (2020). Viscoelasticity in Simple Indentation-Cycle Experiments: A Computational Study. Sci. Rep..

[21] Efremov Y.M., Wang W.H., Hardy S.D., Geahlen R.L., Raman A. (2017). Measuring Nanoscale Viscoelastic Parameters of Cells Directly from AFM Force-Displacement Curves. Sci. Rep..

[22] (2021). Biological Evaluation of Medical Devices—Part 12: Sample Preparation and Reference Materials.

[23] Burggren W., Antich M.R. (2020). Angiogenesis in the Avian Embryo Chorioallantoic Membrane: A Perspective on Research Trends and a Case Study on Toxicant Vascular Effects. J. Cardiovasc. Dev. Dis..

[24] Mohammed Mohammed A.H., Shariff K.A., Wahjuningrum D.A., Bakar M.H.A., Mohamad H. (2023). A Comprehensive Review of the Effects of Porosity and Macro- and Micropore Formations in Porous β-TCP Scaffolds on Cell Responses. J. Aust. Ceram. Soc..

[25] Kim M.H., Tan S.Y., Yamahara K., Kino-oka M. (2023). An in Vitro Culture Platform to Study the Extracellular Matrix Remodeling Potential of Human Mesenchymal Stem Cells. Acta Biomater..

[26] Offeddu G.S., Ashworth J.C., Cameron R.E., Oyen M.L. (2016). Structural Determinants of Hydration, Mechanics and Fluid Flow in Freeze-Dried Collagen Scaffolds. Acta Biomater..

[27] Yu H.S., Kim J.J., Kim H.W., Lewis M.P., Wall I. (2016). Impact of Mechanical Stretch on the Cell Behaviors of Bone and Surrounding Tissues. J. Tissue Eng..

[28] Kohli N., Sawadkar P., Ho S., Sharma V., Snow M., Powell S., Woodruff M.A., Hook L., García-Gareta E. (2020). Pre-Screening the Intrinsic Angiogenic Capacity of Biomaterials in an Optimised Ex Ovo Chorioallantoic Membrane Model. J. Tissue Eng..

[29] Kanellopoulou V., Xanthopoulos A., Mikelis C.M., Papadimitriou E. (2023). Collagens and Collagen-Degrading Enzymes in the Regulation of Angiogenesis. Biol. Extracell. Matrix.

[30] Roura S. (2013). In Vitro Characterization of the Molecular Machinery Regulating Umbilical Cord Blood Mesenchymal Stem Cell Angiogenesis: A Step Towards Multipotent Stem Cell Therapy for Vascular Regeneration. J. Stem Cell Res. Ther..

[31] Merckx G., Tay H., Lo Monaco M., Van Zandvoort M., De Spiegelaere W., Lambrichts I., Bronckaers A. (2020). Chorioallantoic Membrane Assay as Model for Angiogenesis in Tissue Engineering: Focus on Stem Cells. Tissue Eng. Part B Rev..

[32] Alvarez M.M., Liu J.C., Trujillo-de Santiago G., Cha B.H., Vishwakarma A., Ghaemmaghami A.M., Khademhosseini A. (2016). Delivery Strategies to Control Inflammatory Response: Modulating M1–M2 Polarization in Tissue Engineering Applications. J. Control. Release.

[33] Kulakov A., Kogan E., Brailovskaya T., Vedyaeva A., Zharkov N., Krasilnikova O., Krasheninnikov M., Baranovskii D., Rasulov T., Klabukov I. (2021). Mesenchymal Stromal Cells Enhance Vascularization and Epithelialization within 7 Days after Gingival Augmentation with Collagen Matrices in Rabbits. Dent. J..

[34] Korkmaz B., Balli U. (2021). Clinical Evaluation of the Treatment of Multiple Gingival Recessions with Connective Tissue Graft or Concentrated Growth Factor Using Tunnel Technique: A Randomized Controlled Clinical Trial. Clin. Oral Investig..

[35] Yaghobee S., Rouzmeh N., Taheri M., Aslroosta H., Mahmoodi S., Mohammadnejad Hardoroodi M., Soleimanzadeh Azar P., Khorsand A. (2021). Evaluation of Topical Erythropoietin Application on the Healing Outcome of Gingival Graft Recipient Site; a Randomized Controlled Clinical Trial. BMC Oral Health.

[36] Elangovan R., Nallathambi K., Maganti D.C., Maganti A., Soman D., Diwekar N. (2024). Comparison of Root Coverage Procedure Done with and without Loupes along with CGF-A Clinical Study. J. Pharm. Bioallied. Sci..

[37] Sanchez N., Vignoletti F., Sanz-Martin I., Coca A., Nuñez J., Maldonado E., Sanz-Esporrin J., Hernando-Pradíes I., Santamaría S., Herrera D. (2022). Cell Therapy Based on Gingiva-Derived Mesenchymal Stem Cells Seeded in a Xenogeneic Collagen Matrix for Root Coverage of RT1 Gingival Lesions: An In Vivo Experimental Study. Int. J. Mol. Sci..

[38] Bekić M., Radanović M., Ðokić J., Tomić S., Eraković M., Radojević D., Duka M., Marković D., Marković M., Ismaili B. (2022). Mesenchymal Stromal Cells from Healthy and Inflamed Human Gingiva Respond Differently to Porphyromonas Gingivalis. Int. J. Mol. Sci..

[39] Hu Y., Wang Z., Fan C., Gao P., Wang W., Xie Y., Xu Q. (2023). Human Gingival Mesenchymal Stem Cell-Derived Exosomes Cross-Regulate the Wnt/β-Catenin and NF-ΚB Signalling Pathways in the Periodontal Inflammation Microenvironment. J. Clin. Periodontol..

