# Supplementary material for: From Bench to Chairside: Collagen Scaffolds in Combination with Mesenchymal Stromal Cells for Gingival Augmentation
Source: Dent J (Basel). 2025 Oct 8;13(10):460. doi: 10.3390/dj13100460 (PMC12563031; doi:10.3390/dj13100460)
Supplement: Supplementary file 1 [file dentistry-13-00460-s001.zip › dentistry-3808038-supplementary.pdf]

Supplementary Materials

Supplementary Table S1. Porosity analysis.

Matrix type	Total porosity, %	Open porosity, %	Closed porosity, %
Fibro-Gide	78.5	78.57	0.02
FibroMatrix	64.3	64.31	0.11
Mucoderm	33.2	33.15	0.14

Supplementary Table S2. Mechanical parameters of the collagen sponges estimated from microindentation experiments (mean ± SD).

Parameter	Fibro-Gide	FibroMATRIX	Mucoderm
Young's modulus, kPa	12 ± 4	7 ± 4	50 ± 10
Instantaneous modulus, kPa	14 ± 4	8 ± 4	60 ± 14
Long-term modulus, kPa	12 ± 3	7 ± 3	45 ± 8
Relaxation time, s	7.4 ± 1.7	5 ± 2	3.6 ± 2.1

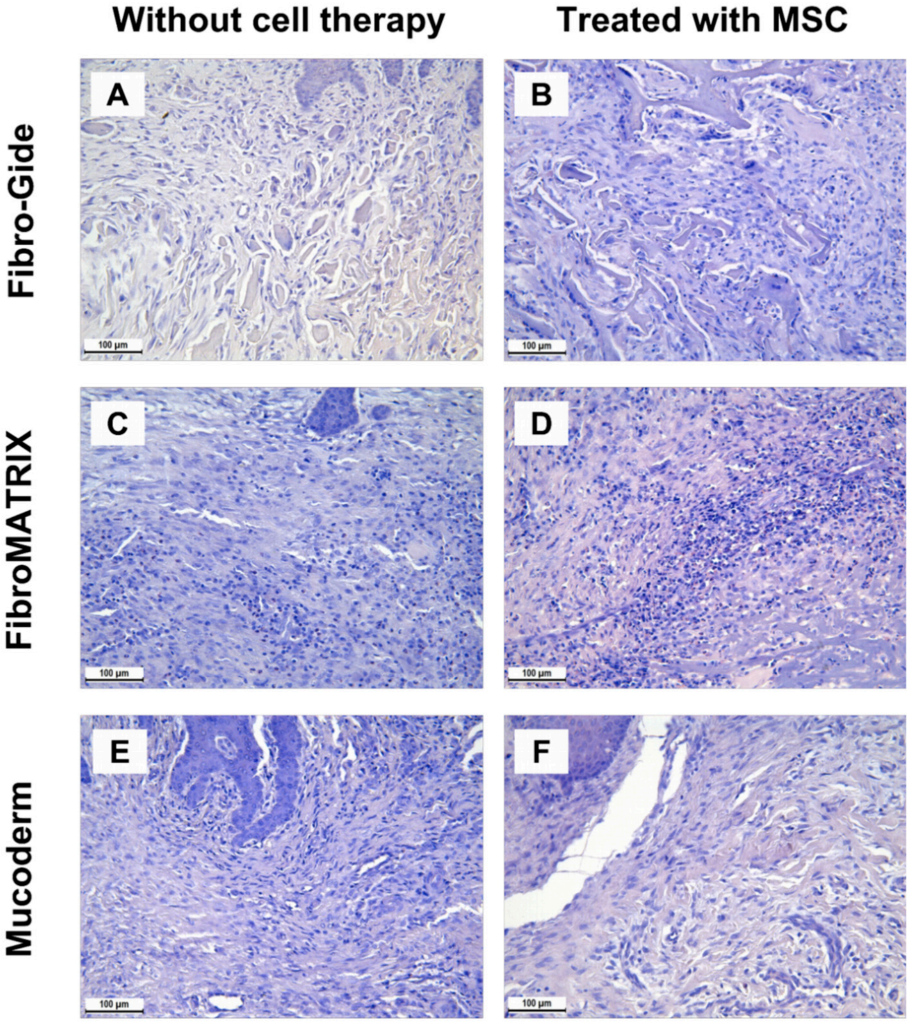

Supplementary Figure S1. Morphological analysis of rabbit gingival tissues at sites of implantation of Fibro-Gide, FibroMatrix, and Mucoderm, with and without cell therapy, hematoxylin, and eosin; magnification ×200.

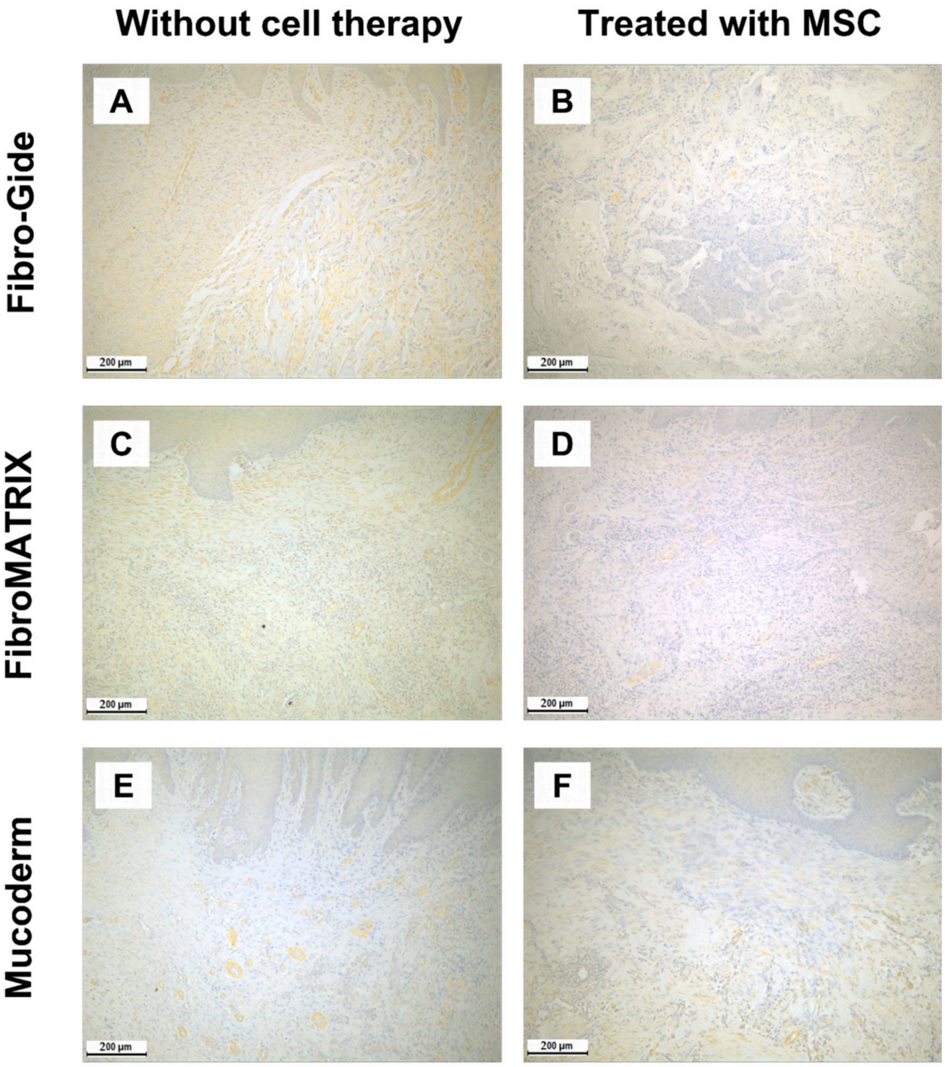

Supplementary Figure S2. Morphological analysis of rabbit gingival tissues at sites of implantation of Fibro-Gide, FibroMatrix, and Mucoderm with and without cell therapy, immunohistochemical reaction with antibodies against α-smooth muscle actin; magnification ×100.
